# Mapping the Landscape of Tau Post-translational Modifications and Kinase-Specific Phosphorylation Signatures by Orthogonal CZE-MS/MS and NanoRPLC-MS/MS

**DOI:** 10.1021/acs.jproteome.6c00539

**Published:** 2026-10-13

**Authors:** Reyhane Tabatabaeian Nimavard, Seyed Amirhossein Sadeghi, Jorge A Colón Rosado, Yifan Yue, Fei Fang, Hsiao-Tien Chien Hagar, Min-Hao Kuo, Liangliang Sun

**Affiliations:** † Department of Chemistry, 3078 Michigan State University, 578 S Shaw Lane, East Lansing, Michigan 48824 United States; ‡ Department of Biochemistry and Molecular Biology, 115977 Michigan State University, 603 Wilson Road, East Lansing, Michigan 48824, United States

**Keywords:** Alzheimer’s disease, Tau, Phosphorylation, Kinase specificity, CZE-MS/MS, nanoRPLC-MS/MS

## Abstract

Hyperphosphorylation of tau is central to the development and progression of Alzheimer’s disease (AD) and other tauopathies. How different kinases and their phosphorylated tau products, as well as additional post-translational modifications (PTMs) of tau, contribute to AD is a critical yet poorly understood question. We created a comprehensive landscape of PTMs (i.e., phosphorylation, acetylation, and methylation) of tau across isoforms (2N4R, 1N4R, and 0N3R) and kinases (GSK3β, TTBK1, and DYRK1A) using complementary capillary zone electrophoresis (CZE)-tandem mass spectrometry (MS/MS) and nanoflow reversed-phase liquid chromatography (nanoRPLC)-MS/MS. Kinase-treated 2N4R (Tau-441) yielded 65 phosphorylation sites in total, consisting of 31 shared sites and additional kinase-specific subsets; 15 phosphosites were observed only in kinase-treated samples. All kinases generated the AT8 (Ser202/Thr205) and PHF-1 (Ser396/Ser404) epitopes. Across all conditions and isoforms, we identified 76 unique phosphorylation sites on Tau-441. Orthogonal separations provided near-complete Tau-441 sequence coverage (CZE: 96.3 ± 2.5%; RPLC: 95.9 ± 2.8%; n = 12 runs each) with phosphorylation sites detected uniquely by either platform. In addition, 49 Lys/Arg residues were acetylated and/or methylated (including K174, K274, and K281). This work revealed convergent and kinase-specific tau PTM patterns relevant to AD mechanisms and kinase prioritization. We expect the comprehensive kinase-specific PTM resource of tau generated here will benefit future tau-focused AD research.

## Graphical abstract

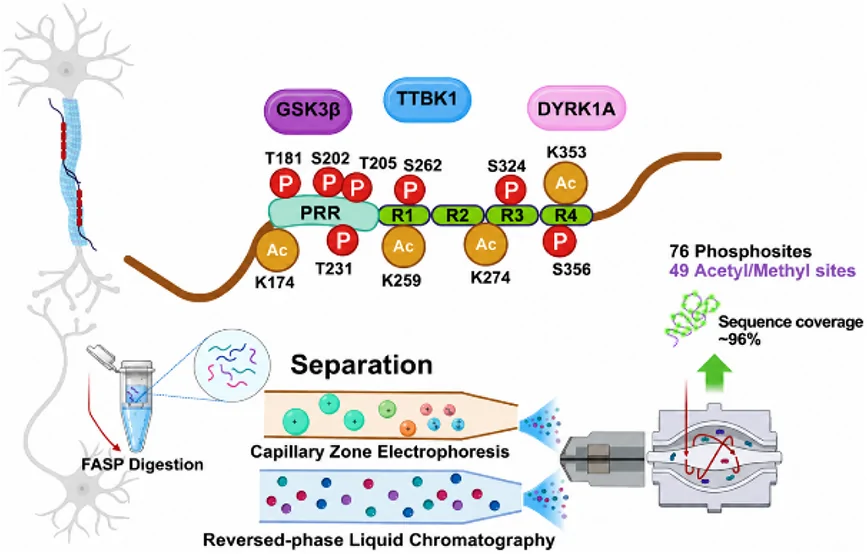

## Introduction

Alzheimer’s disease (AD) is defined pathologically by the accumulation of extracellular amyloid-β plaques and intracellular neurofibrillary tangles (NFTs) composed of tau protein, as first noted by Alois Alzheimer over a century ago.^1–3^ Of these two hallmark pathologies, tau pathology bears a particularly strong relationship to neurodegeneration and clinical symptoms, with the burden of NFTs correlating more closely with cognitive decline and disease severity than Aβ senile plaque.^3–5^ This central role of tau in AD progression has driven intense interest in tau as both a therapeutic target and a disease biomarker.^6,7^ In fact, recent advances in fluid biomarkers have capitalized on disease-associated tau modifications.^8–10^ For example, elevated plasma tau phosphorylated at threonine-217 (p-tau217) accurately distinguishes AD from other dementias with remarkable sensitivity and specificity.^11,12^ Likewise, tau phosphorylated at threonine-231 (p-tau231) in plasma has emerged as an early indicator of AD pathology, even before the onset of overt dementia.^12,13^ Such findings underscore that aberrant tau phosphorylation is not only a hallmark of neuropathology but also a reflection of the disease process measurable in patients’ biofluids.^14^

Tau is a large microtubule-associated protein encoded by the *MAPT* gene, which produces six isoforms (352–441 amino acids) in the adult human brain through alternative splicing.^15^ These isoforms differ by the presence of three or four microtubule-binding repeat domains (3R vs 4R tau) and 0, 1, or 2 N-terminal inserts, and all six isoforms are found in normal brain tissue.^8,15^ This isoform diversity, combined with tau’s highly repetitive and proline-rich sequences, complicates proteomic analyses, as many peptides are shared between isoforms or occur in low-complexity regions, making full sequence coverage and site-specific mapping challenging.^16^ Moreover, tau undergoes numerous post-translational modifications (PTMs) beyond phosphorylation that can modulate its function and aggregation behavior.^17,18^ Notably, tau acetylation at certain lysine residues is a disease-associated PTM. It can be detected even in early AD and tends to intensify as the disease progresses, potentially exacerbating tau fibrillization and toxicity.^19,20^ In aggregate, the complexity of tau’s isoforms and PTMs presents a major analytical challenge but also hints at the rich regulatory landscape through which tau contributes to neurodegeneration.^21^

A variety of protein kinases have been implicated in driving tau hyperphosphorylation in AD. Glycogen synthase kinase-3β (GSK3β), for example, has long been recognized as a key tau kinase whose overactivation in the AD brain correlates with the aberrant tau phosphorylation and NFT formation.^22–24^ Other kinases, including cyclin-dependent kinase 5 (CDK5), casein kinase 1, and stress-activated kinases, also phosphorylate tau at disease-relevant sites.^25,26^ More recently, kinases such as tau tubulin kinase 1 (TTBK1) and dual-specificity tyrosine-phosphorylation regulated kinase 1A (DYRK1A) have also attracted attention. TTBK1 is a neuron-specific kinase that phosphorylates tau and is significantly upregulated in AD brains.^27,28^ Genetic studies have even linked variations in the *TTBK1* gene to an increased risk of late-onset AD.^28,29^ DYRK1A, on the other hand, is overexpressed in Down syndrome (which confers early AD pathology) and has been shown to phosphorylate tau at multiple sites.^30–32^ The involvement of these distinct kinases raises the critical question of whether each enzyme leaves a unique “fingerprint” on tau, that is, a specific pattern of tau phosphorylation that might define particular stages or forms of AD. Identifying such kinase-specific phosphorylation signatures is the central motivation of this study.^33^ By deciphering which phosphorylation sites are attributable to which kinases, we aim to contribute to untangle the complex kinase-tau network and provide more useful information on how each kinase’s activity contributes to the progression of tau pathology in AD.

To this end, we integrated orthogonal separation techniques, capillary zone electrophoresis-tandem MS (CZE-MS/MS), alongside conventional reversed-phase liquid chromatography-MS/MS (RPLC-MS/MS), to achieve deep coverage of tau PTMs. Orthogonal separation means that CZE and RPLC resolve peptides based on different physicochemical properties (electrophoretic mobility vs hydrophobicity), thereby greatly increasing the overall proteome coverage. Indeed, recent studies have demonstrated that coupling nanoRPLC fractionation to CZE-MS/MS can identify far more peptides and proteins from limited samples than either method alone, due to the complementary nature of their separation mechanisms and the enhanced sensitivity of CZE-MS/MS.^34^

By leveraging this orthogonal CZE/RPLC-MS/MS platform, we obtain a bird’s-eye yet site-resolved view of tau phosphorylation patterns across the full length of the protein. We then interpret these patterns in the context of specific kinase activities, testing the hypothesis that distinct tau kinases preferentially target specific residue subsets.

## Experimental Section

### Materials and Reagents

All standard chemicals were purchased from Sigma-Aldrich (St. Louis, MO) unless otherwise noted, including dithiothreitol (DTT), iodoacetamide (IAA), ammonium bicarbonate, and trypsin. LC-MS-grade solvents (water, methanol, formic acid, and acetic acid) were obtained from Fisher Scientific (Pittsburgh, PA). Acrylamide monomer was purchased from Acros Organics (Fair Lawn, NJ). Fused-silica capillaries (50 μm inner diameter × 360 μm outer diameter) were acquired from Polymicro Technologies (Phoenix, AZ).

### Tau Protein Samples

#### Commercial Tau-441

Recombinant human tau protein (2N4R isoform, full-length 441 amino acids with a C-terminal His-tag) was purchased from Sino Biological (Cat. #30132-H07E, Beijing, China). In addition, three kinase-phosphorylated Tau-441 preparations were obtained as frozen solutions from SignalChem (via Sino Biological). These included GSK3β-phosphorylated Tau-441 (Cat. #T08–50FN), TTBK1-phosphorylated Tau-441 (Cat. #T08–50ON), and DYRK1A-phosphorylated Tau-441 (Cat. #T08–50RN). According to the certificates of analysis, these kinase-phosphorylated Tau-441 proteins were generated by coexpression of recombinant human tag-free Tau-441 with the corresponding kinase in *E. coli*, followed by additional in vitro phosphorylation prior to final chromatography purification. Throughout this manuscript, references to 2N4R Tau-441 denote the commercially sourced recombinant Tau-441 preparations, including the untreated control and the GSK3β-, TTBK1-, and DYRK1A-phosphorylated commercial samples. According to the manufacturer, the GSK3β- and TTBK1-phosphorylated tau samples were formulated in 50 mM Tris–HCl (pH 7.5), 150 mM NaCl, 1 mM DTT, 10% glycerol, whereas the DYRK1A-phosphorylated tau was supplied in 50 mM Tris–HCl (pH 7.5), 150 mM NaCl, 0.25 mM DTT, 0.1 mM PMSF, 25% glycerol. All tau protein samples were stored at −80 °C in small aliquots to minimize freeze–thaw cycles and handled according to the manufacturer’s recommendations.

#### PIMAX Tau Expression and Purification

Tau isoforms 0N3R and 1N4R were expressed in *E. coli* BL21-CodonPlus cells using the PIMAX system, adapted from the method of Sui et al.^26,35^ In this system, tau was produced under three conditions: control (tau expressed without kinase coexpression), CDK5-phosphorylated, and GSK-3-phosphorylated. For the phosphorylation conditions, tau was coexpressed with either CDK5 or GSK-3, together with Fos/Jun leucine-zipper recruitment to enhance kinase-tau association and promote in vivo phosphorylation. BL21-CodonPlus cells harboring the appropriate PIMAX tau plasmids were induced with IPTG for recombinant protein expression. Cell pellets were lysed, clarified, and heat-treated to exploit the heat stability of tau, followed by TEV protease cleavage to remove fusion elements. The released tau proteins were then clarified, concentrated using a 30 kDa MWCO filter, buffer-exchanged into 20 mM Tris-HCl (pH 7.4), 100 mM NaCl, supplemented with 10% glycerol, and stored at −80 °C until further analysis.

### Proteolytic Digestion and Sample Preparation

Prior to proteomic analysis, tau protein samples underwent bottom-up digestion using a filter-aided sample preparation (FASP) workflow.^36^ Approximately 10 μg of protein from each sample was incubated at 37 °C for 1 h before reduction and alkylation. Disulfide bonds were then reduced with 10 mM DTT at 37 °C for 1 h, followed by alkylation with 10 mM IAA for 30 min at room temperature in the dark. Residual IAA was quenched by adding 10 mM DTT and incubating for 20 min at room temperature. The protein solution was then transferred to a 30 kDa MWCO ultrafiltration unit (assembled in a horizontal orientation) as described by Wiśniewski et al.,.^36^ allowing buffer exchange and digestion on the filter. The filter sample was washed five times with 50 mM ammonium bicarbonate to remove small molecules. Trypsin was added to the retentate (enzyme-to-substrate ratio of ∼ 1:30, w/w), and the mixture was incubated overnight at 37 °C to digest proteins. The resulting tryptic peptides were collected by centrifugation of the filter unit. For capillary zone electrophoresis analysis, peptide samples were adjusted to a concentration of ∼ 0.4 mg/mL. For nanoLC–MS analysis, peptide solutions were prepared at 0.10 mg/mL (and further diluted to ∼ 0.03 mg/mL).

### CZE-MS/MS Analysis

Capillary zone electrophoresis-tandem mass spectrometry (CZE-MS/MS) was performed on a CESI 8000 Plus capillary electrophoresis system (Beckman Coulter) coupled to a Thermo Fisher Orbitrap Exploris 480 mass spectrometer. The CE-MS interface employed an in-house electrokinetically pumped sheath-flow nanoelectrospray interface, as described previously.^37^ Linear polyacrylamide (LPA)-coated fused-silica capillaries (50 μm i.d. × 360 μm o.d., 100 cm length) were used to minimize electroosmotic flow.^38^ The outlet end of the separation capillary was etched with hydrofluoric acid to a ∼100 μm diameter.^39^ A glass nanospray emitter with a 30–35 μm orifice was filled with sheath liquid (0.2% v/v formic acid, 10% v/v methanol). The background electrolyte was 5% v/v acetic acid, and separations were carried out at +30 kV. Approximately 70 nL of sample (∼0.4 mg/mL peptide digest) was injected per run. Between runs, a high-voltage pressure flush with fresh buffer was performed to rinse the capillary and reduce carryover. Each sample condition was analyzed in three technical replicates.

The Orbitrap Exploris 480 mass spectrometer was operated in positive-ion mode with nanoelectrospray ionization and data-dependent acquisition (DDA). The spray voltage was set to 2.0 kV, the ion transfer tube temperature was 320 °C, and the RF lens was 50%. Full MS scans were acquired in the Orbitrap at a resolving power of 60,000 (at *m*/*z* 200) over the *m*/*z* range of 300–1600, with one microscan, profile-mode detection, an AGC target of 300%, and a maximum injection time of 50 ms. Advanced peak determination was enabled, monoisotopic precursor selection was set to peptide mode, and precursor selection used an intensity threshold of 5.0 × 10^4^. Only ions with charge states 2–7 were selected for fragmentation; singly charged and undetermined-charge ions were excluded.

MS/MS spectra were acquired using HCD with a normalized collision energy of 28%, a 2.0 *m*/*z* isolation window, and no isolation offset. Product ion spectra were collected in the Orbitrap at a resolving power of 60,000 (at *m*/*z* 200) over the *m*/*z* range of 200–2000, with one microscan, centroid-mode detection, an AGC target of 100%, and a maximum injection time of 100 ms. A Top 10 DDA method was used, with up to 10 MS/MS scans acquired per full MS scan. Dynamic exclusion was enabled for 15 s with a mass tolerance of ± 10 ppm. Isotope peaks were excluded, and only one charge state per precursor was fragmented in each cycle.

### RPLC-MS/MS Analysis

For comparison, tryptic tau digests were also analyzed by nanoflow LC–MS/MS. Peptide separations were performed on an EASY-nLC 1200 system (Thermo Fisher) coupled to the Orbitrap Exploris 480 with a nanoelectrospray source. About 30 ng of peptides was loaded onto a home-packed C18 capillary column (75 μm i.d. × 360 μm o.d., 20 cm in length, ReproSil-Pur 120 C18-AQ, 1.9 μm, 120 Å) and separated in a 70 min run. The column was equilibrated in solvent A (0.1% formic acid in water), and peptides were eluted with a linear gradient of 8–55% solvent B (0.1% formic acid in acetonitrile) over 50 min, then 55–100% B over 5 min, at 200 nL/min. The column was flushed at 100% B for an additional 15 min before re-equilibration. Orbitrap Exploris 480 was operated with the same DDA parameters as in the CZE-MS/MS analysis.

### Data Analysis

Raw files were processed in Thermo Proteome Discoverer 2.2 using the Sequest HT search engine. Controls, CDK, GSK3β, TTBK1, and DYRK1A samples were searched separately, and the three technical replicates for each condition were searched independently and merged afterward for condition-level interpretation. For each tau isoform, database searches were performed against a FASTA database containing the corresponding tau isoform sequence together with the *Escherichia coli* proteome, consistent with the previously reported workflow.^34^ Trypsin was specified as the protease with up to three missed cleavages allowed. The precursor ion mass tolerance was set to 10 ppm, and the fragment ion mass tolerance to 0.02 Da. Peptide lengths of 6–144 amino acids were permitted. A target-decoy strategy implemented through Percolator was used for confidence estimation, and peptide-spectrum matches (PSMs) were filtered at 1% false discovery rate (FDR) for final reported identifications. Carbamidomethylation of cysteine was set as a fixed modification. Variable modifications, with a maximum of five dynamic modifications per peptide, included oxidation (Met), acetylation (Lys, Arg), phosphorylation (Ser, Thr, Tyr), succinylation (Lys) (Succinylation was included in the exploratory search space; however, not confidently localized succinylation sites passed the reporting criteria, and it was therefore not analyzed further.), and methylation (Lys, Arg). Within Proteome Discoverer 2.2, the Spectrum Selector node preprocessed each MS/MS spectrum by retaining the 12 most intense fragment peaks in each 100 Da window before Sequest HT database searching. Site localization was performed in Proteome Discoverer using the ptmRS node. Localization probabilities were calculated for all identified modified peptides, and only phosphorylation sites with localization probability ≥ 75% were retained as confidently localized. After independent searching of the three technical replicates, replicate results were merged by condition, and a phosphosite was considered present in a condition when supported by at least one confidently localized observation. To provide a limited quantitative comparison of representative shared phosphopeptides, MS1 intensities were manually extracted from the raw CZE-MS data for phosphopeptides containing T212, S305, S324, and S352 across the three technical replicates of untreated, GSK3β-, DYRK1A-, and TTBK1-treated 2N4R tau. Each kinase-treated condition was compared with the untreated control using a two-tailed Welch’s unpaired *t* test (n = 3 technical replicates per condition).

## Results and Discussion

### Overview, Counting Rules, and Study Design

Phosphorylation sites were counted as unique residue positions mapped to Tau-441 (2N4R) numbering, and the reported site lists represent the union of CZE-MS/MS and nanoRPLC-MS/MS identifications that pass 1% PSM FDR and have ptmRS localization probability ≥75%. For all conditions analyzed in this study, each sample was acquired in three technical replicates per platform, and site lists were compiled as the union across runs. The data sets included the commercial 2N4R kinase comparison (untreated, GSK3β-, TTBK1-, and DYRK1A-treated samples) as well as 1N4R and 0N3R samples analyzed under control, CDK, and GSK3β conditions. Across all isoforms and conditions evaluated in this work, we identified 76 distinct phosphorylation sites and 49 distinct acetylation or methylation sites at the residue level. Replicate-level sequence coverage values for the CZE-MS/MS and nanoRPLC-MS/MS data sets are summarized in the Supporting Information.

Figure 1 illustrates the overlap and uniqueness of phosphorylation sites on the 2N4R tau isoform (441 residues) when phosphorylated by GSK3β, DYRK1A, or TTBK1. In total, 65 distinct phosphosites were identified across the three kinases in our analysis. GSK3β, TTBK1, and DYRK1A each phosphorylated 51, 52, and 41 sites, respectively. A core set of 31 sites was shared among all three kinases, indicating convergent targeting of many tau residues (Figure 1B). These commonly phosphorylated sites span the proline-rich region and microtubule-binding repeats and include several well-known Alzheimer’s disease (AD) phospho-epitopes,^40^ Table S1. For example, the AT8 epitope (tau pSer202/pThr205) ^41^was generated by all three kinases, consistent with reports that multiple kinases (including GSK3β and CDK5) can phosphorylate this site and that phosphorylation at Ser202/Thr205 contributes to tau filament formation.^42^ Similarly, the PHF-1 epitope (tau pSer396/pSer404)^43,44^ was found in all three kinase data sets; these two C-terminal sites are commonly hyperphosphorylated in AD paired helical filaments, and notably phosphorylation at Ser396 by GSK3β or CDK5 is known to destabilize microtubules.^41^ To further contextualize these findings, Table S1 details well-characterized tau phosphorylation sites linked to aggregation in AD, including an overview of hallmark phospho-epitopes, including AT8, AT100, AT180, and PHF-1.

**Figure 1:**
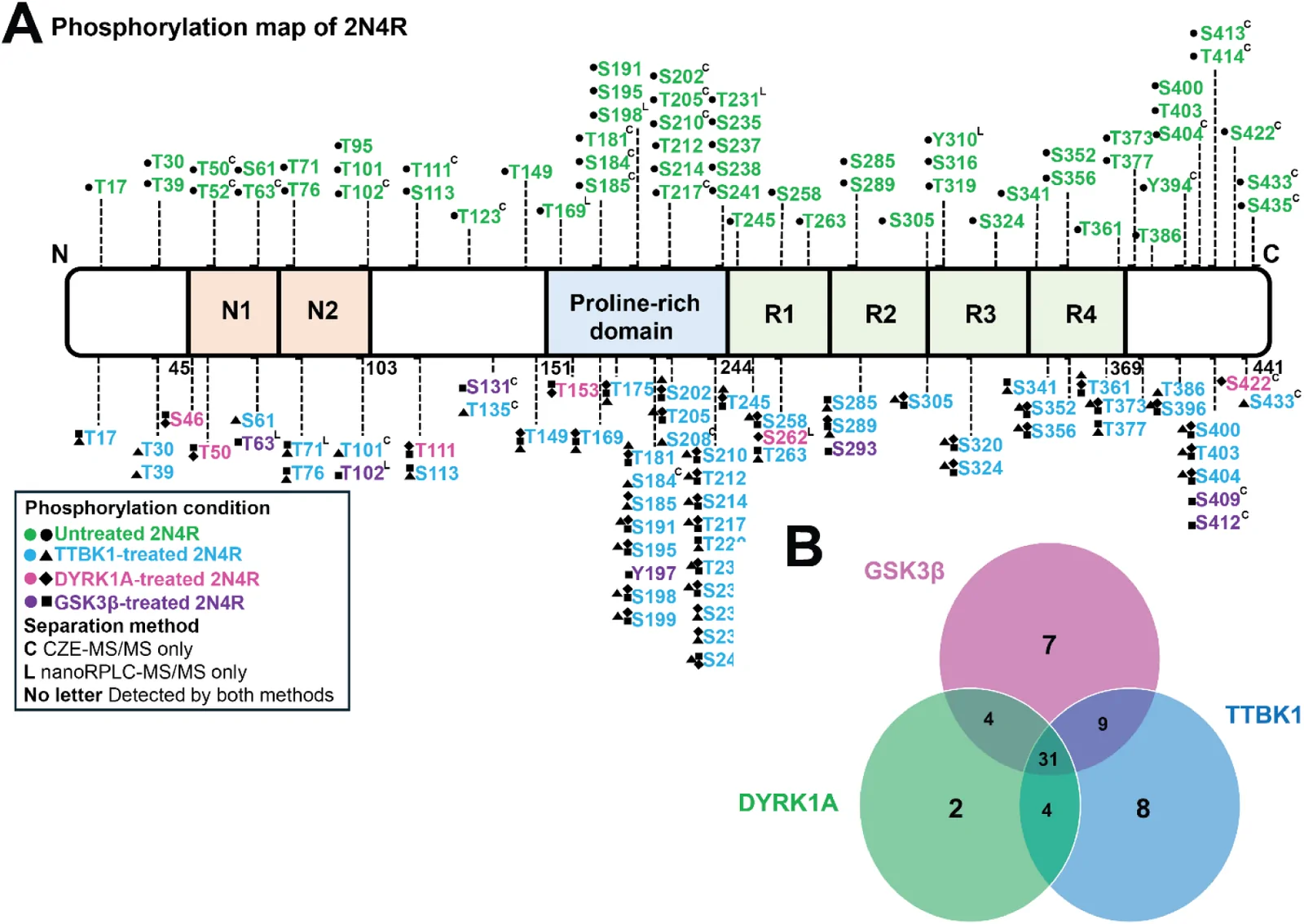
Residue-level phosphorylation map of 2N4R tau across kinase treatments. (A) Phosphosites are encoded by colored labels and black symbols: green circles, untreated; blue triangles, TTBK1-treated; orange diamonds, DYRK1A-treated; and magenta squares, GSK3β-treated 2N4R. Multiple symbols indicate sites shared among conditions. Superscripts C and L indicate sites detected exclusively by CZE-MS/MS and nanoRPLC-MS/MS, respectively; no superscript indicates detection by both. Tau domains are annotated. (B) Venn diagram showing shared and kinase-specific phosphosites: 31 were common to all three kinases, whereas 7, 8, and 2 were unique to GSK3β, TTBK1, and DYRK1A, respectively.

Beyond these, the shared phosphosites among kinases include many proline-directed Ser/Thr-Pro motifs (e.g., Thr181, Ser202, Thr212, Ser235) as well as residues in the repeat domains (e.g., Ser258, Ser324, Ser352, Ser356), Figure 1A, aligning with the observation that phosphorylation in tau’s repeat region strongly diminishes its microtubule binding affinity.^45^ Tau contains four conserved KXGS motifs in the MTBR (Ser262, Ser293, Ser324, and Ser356), Figure 1A, and phosphorylation at these sites is known to weaken tau-microtubule interactions and promote tau detachment from the microtubule lattice.^46,47^ Also, Ser262, a critical site in the first repeat, was identified as phosphorylated in the DYRK1A reaction, notably, only DYRK1A among the three kinases phosphorylated Ser262 in our assays, suggesting a unique capacity of DYRK1A to modify this microtubule-binding motif. In contrast, Ser409, a site in the distal C-terminal tail, was exclusively phosphorylated by GSK3β. Phosphorylation at Ser409 is barely detectable in normal adult brain but emerges prominently in AD tau pathology, and our finding that GSK3β alone could produce pSer409 underscores GSK3β’s potent and disease-relevant activity on tau.

To complement the residue-level presence/absence comparison, we examined the MS1 intensities of four representative phosphopeptides containing T212, S305, S324, and S352 across the four conditions (untreated, GSK3β, DYRK1A, or TTBK1 treated), Figure S2. The raw phosphopeptide intensity data are listed in Supporting Information II. We identified these four peptides in all four conditions. Although we identified the phosphopeptides in all conditions, their intensities varied substantially, indicating condition-dependent phosphorylation extent at specific sites. For example, the pT212 peptide showed substantially higher MS1 signal in the DYRK1A-treated sample. The pS305 peptide showed significantly lower MS1 intensity in the TTBK1-treated sample than in the other three samples. These data highlight the importance of studying the extent of tau phosphorylation to accurately understand how tau phosphorylation affects AD progression, which will be the focus of our future study.

Regarding a distinctive set of unique phosphorylation sites not shared with the others, GSK3β uniquely phosphorylated 7 sites on tau: Thr63, Thr102, Ser131, Tyr197, Ser293, Ser409, and Ser412. TTBK1 phosphorylated 8 sites that were not observed with the other enzymes: Thr30, Thr39, Ser61, Thr101, Thr135, Ser184, Thr386, and Ser433. DYRK1A, which overall showed more limited substrate coverage, uniquely targeted two sites: Ser262 and Ser422. Several kinase-specific sites were newly identified in this analysis and, to our knowledge, have not been widely reported in prior studies as tau phosphosites generated by these specific kinases. In particular, the sites in tau’s extreme N-terminus Thr30, Thr39, and Ser61 (TTBK1-specific) and Thr63 (GSK3β-specific), as well as C-terminal tail sites like Thr386 and Ser433 (TTBK1-specific), were only detected with the improved methodology used here. The identification of these sites may provide new insights into kinase-targeting preferences. For instance, TTBK1’s unique sites at the far N- and C-termini (e.g., Ser61, Ser433) suggest that this brain-specific kinase can phosphorylate regions of tau that are less favored by the other kinases. Likewise, GSK3β’s unique modification of Ser412 (adjacent to the PHF-1 epitope) and DYRK1A’s exclusive Ser262 mark reinforce that each kinase has preferred target motifs or conformational requirements that result in selective site phosphorylation.

The overlapping subsets of phosphosites between pairs of kinases are also informative. GSK3β and TTBK1 showed the largest two-kinase overlap, sharing 9 sites beyond the common core (Thr17, Thr71, Thr76, Ser113, Thr220, Thr263, Ser285, Ser341, and Thr377). GSK3β and DYRK1A shared 4 additional sites (Ser46, Thr50, Thr111, and Thr153) that TTBK1 did not phosphorylate. TTBK1 and DYRK1A in turn shared 4 sites (Ser185, Ser208, Ser237, and Ser238) that were not hit by GSK3β. These patterns suggest that GSK3β and TTBK1 have broader and somewhat convergent specificity, whereas DYRK1A phosphorylates a more restricted set that partially overlaps with each of the other two. The absence of certain sites in one kinase’s profile often correlates with known biochemical preferences. For example, priming phosphorylation is a well-established requirement for GSK3β. GSK3β alone is less efficient at sites such as Thr181 or Thr231 in the absence of a preexisting phosphate at the neighboring +4 position.^48^ DYRK1A is known to prime tau at Thr212 (an SP/TP site in the proline-rich region). In our single-kinase experiments, Ser208 was phosphorylated by TTBK1 but not by GSK3β, consistent with the idea that GSK3β requires Thr212 (or another nearby site) to be primed before phosphorylating Ser208. DYRK1A’s unique ability to hit Ser262 (a non-Proline motif in the repeats) and Ser422 (in the tail) also points to its role as an initiating kinase for certain sites. Interestingly, although TTBK1 has been implicated as a major kinase for Ser422 in cells and in vivo,^29^ our data did not detect Ser422 phosphorylation in TTBK1 reactions; it appeared only with DYRK1A. This discrepancy should stem from the different expression systems of tau and kinases in these two studies (*E. coli* vs brain). Nonetheless, TTBK1 did mark several other disease-relevant sites (e.g., Ser208, Ser238, and Ser241) that overlap with DYRK1A’s profile, supporting the notion that both kinases contribute to pathological tau hyperphosphorylation in overlapping regions.^30^ GSK3β, often considered a central tau kinase in AD, covered the greatest number of sites, including almost all hallmark epitopes (AT8, AT100, PHF-1, etc.) and uniquely added certain late-stage sites like pSer409 (detected by the AD-specific antibody PG-5).^49,50^ Also, comparison with the curated tau phosphorylation compendium summarized by Hanger and colleagues showed substantial agreement between our experimentally identified phosphorylation sites and previously reported tau phosphosites.^51^ Taken together, these results highlight that, while all three kinases can phosphorylate tau at many common sites related to microtubule binding and AD epitopes, each kinase also has a unique activity signature. This specificity could be due to differences in consensus sequence recognition, priming requirements, or structural accessibility of tau regions to each kinase. Complete residue-level phosphosite inventories for the 2N4R kinase comparison, together with kinase-overlap annotations and literature-context tables, are provided in Supporting Information II.

Methodological improvements in our phosphosite mapping underpin some of these new insights. In this analysis, we combined capillary zone electrophoresis (CZE)-MS/MS with traditional reversed-phase LC­(RPLC)-MS/MS to achieve deeper coverage of tau phosphopeptides. The use of CZE, which offers high separation efficiency for peptides, substantially increased our ability to detect low-abundance or previously coeluting phosphopeptides. In a recent study on a shorter tau isoform (0N3R), adding CZE-MS yielded ∼ 30% more phosphosites than RPLC-MS alone, in line with our experience here with full-length 2N4R tau.^34^ For example, peptides containing Thr101 and Ser433 (highly hydrophilic regions at tau’s termini) were more readily identified with CZE, explaining why new N-terminal and C-terminal sites appear in the TTBK1 data set now. By leveraging both separation techniques, we minimized analytical blind spots and uncovered additional phosphosites, thereby refining the kinase-specific profiles.

### Lysine and Arginine Acetylation and Methylation across Tau Isoforms

Our proteomic analysis reveals a diverse array of tau acetylation and methylation sites across all domains of the longest tau isoform (2N4R). We identified 49 lysine or arginine residues carrying PTMs across all three tau isoforms (0N3R, 1N4R, and 2N4R), Figure 2. Many modifications were observed even in tau from control samples (e.g., constitutive methylation of R23 and dual acetylation/methylation of K24 and K44), whereas others emerged only under kinase treatment or in specific isoforms. Notably, several lysines known to be pathologically acetylated in AD (K174, K274, K281)^52^ were absent or barely detectable in normal tau in recent studies, highlighting their disease specificity.^52,53^ Conversely, we identified multiple novel PTMs, particularly in the N- and C-terminal regions, that have not been reported in prior tau studies. Detailed residue-level PTM inventories across 0N3R, 1N4R, and 2N4R tau, including 2N4R-converted site mappings and summary tables of novel or context-specific modifications, are provided in Figure 2 and Supporting Information II. As detailed in Supporting Information I, many of the lysine/arginine modifications we observed (particularly in the N-terminal and repeat domains) are newly identified. The N-terminal “projection” domain harbored numerous PTMs, but nearly all appear to be constitutive or context-specific rather than disease-related. The extreme N-terminus contains marks such as methylated R23 and acetylated K24 and K44, which have no documented role in tau pathology.^54^ Several modifications within the two N-terminal inserts were highly dependent on the isoform and kinase, e.g., K67 methylation only observed in the 1N4R treated by CDK, K67 acetylation only detected in 2N4R treated by GSK3β, and K87 acetylation only identified in the 2N4R treated by GSK3β.^55^ We also identified a cluster of novel acetylation sites in the mid-N-terminus (∼residues 130–143), none of which have been previously reported. One notable exception in this region is Lys150, at the border of the proline-rich domain. K150 has been identified as an acetylation site in human tauopathies (including AD)^53^ and was uniquely acetylated in a recent tauopathy study.^54^ Our detection of K150 acetylation aligns with these reports, confirming it as a bona fide pathological site. Thus, while the N-terminal domain contains numerous lysine acetylation and methylation sites (many newly identified), relatively few known AD-linked acetylation sites map to this region. However, these modifications could still influence tau function by altering local charge distribution and electrostatic interactions, which are known to regulate tau conformation, microtubule binding, and aggregation.

**Figure 2:**
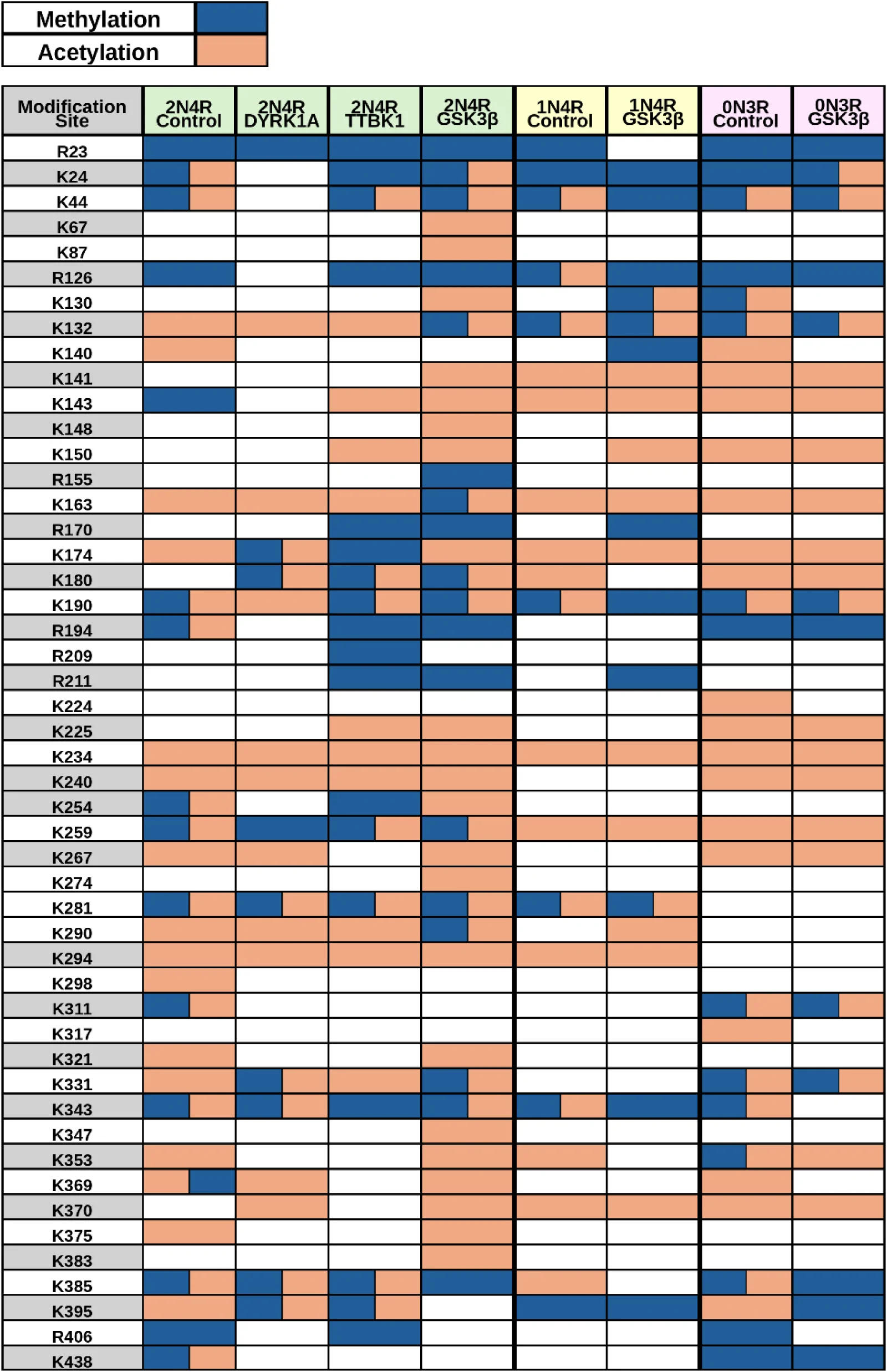
Lysine and arginine acetylation (orange) and methylation (blue) across tau isoforms and kinase treatments. The figure highlights constitutive methylation and acetylation, showing isoform- and kinase-specific patterns in tau.

Tau’s proline-rich domain contains several of the “classical” AD-associated acetylation sites, as well as other regulated PTMs.^56^ The standout modification in this region is Lys174, which was acetylated in our data set under multiple conditions (including control tau and various kinase treatments). This site is well-established as an early pathological acetylation: K174 acetylation is significantly elevated in AD brains and is known to slow tau turnover and induce toxicity when mutated to an acetyl-mimic.^53,57^ Our findings confirm K174 as an acetylation hotspot on tau and reinforce its importance in disease. Adjacent to K174, we observed Lys163 acetylation even in control tau. Notably, K163 has also been reported as one of the major lysines acetylated in AD tau filaments.^58^ In addition to lysine acetylation, multiple arginine methylation sites were identified in this region. R155 methylation was observed (a mark seen in both normal and AD tau^59^), and several other methylation sites (R170, R194, R209, and R211) appear to be novel additions to tau’s PTM profile.

The proline-rich region also exhibited lysine acetylation patterns similar to those observed in prior AD studies.^60^ Lys224 and Lys225 (just C-terminal to the polyproline motifs) were acetylated in our analysis (K224 in untreated 0N tau; K225 in 0N and 2N tau), although neither has been reported previously (novel sites). By contrast, Lys234 and Lys240 were acetylated in almost all samples (especially in 2N4R and 0N3R tau). Both K234 and K240 are known pathological sites: proteomic studies have identified ac-K234 and ac-K240 as major disease-associated marks in AD. Our observation of acetylation at K234 and K240 in control tau indicates that these residues can be modified under normal conditions, although their pronounced acetylation in diseased tau distinguishes them as pathology-linked.^18,61^ Finally, at the junction with the repeat region lies Lys274, a well-characterized pathological hotspot. K274 is among the lysines predominantly acetylated in AD brains and is mechanistically linked to tau fibrillization and toxicity.^52^ Importantly, we did not detect K274 acetylation in normal tau; it was observed only under one *kinase* condition (GSK3β-treated 2N4R tau). The absence of K274 in nonpathological conditions underscores that its acetylation is disease-specific. In AD brains, ac-K274 is abundant^52^ and has been shown to disrupt microtubule binding and enhance aggregation.^62^ In summary, the proline-rich region exhibits a mix of constitutive modifications (e.g., methylated R155) and disease-relevant acetylation (e.g., K163, K174, K234, K240, and K274), as well as several novel sites (not observed in prior studies).^18^ This pattern underscores the central role of the proline-rich region in regulating tau and in the early development of tauopathy.

The repeat domain of tau (comprising four repeats in 2N4R and three repeats in 0N3R) harbors many canonical tauopathy-associated acetylation sites alongside numerous novel marks. Notably, Lys281, a prominent disease-associated site, showed acetylation even in normal brain tau in our study. This suggests that K281 can be modified at basal levels, perhaps serving a physiological role, even though acetylation at this site is far more extensive in tauopathies.^52^ Within the repeats, Lys259 (in the first repeat, R1) stood out as another major site. K259 is among the top acetylation sites in AD tau,^18,63^ and strong acetyl-tau signals at this residue have been observed in diseased tissue. Consistent with this, we detected acetylation (and even some methylation) at K259 in normal tau, indicating that this modification is not exclusively pathological, though it is clearly enriched in disease. Similarly, Lys311 (in R3) is a known pathological acetylation site^18,63^ that we found to be modified (acetylated and methylated) at baseline, suggesting that K311 is a constitutive site that nonetheless becomes part of the pathological acetylation repertoire. Overall, the repeat region contains multiple disease-associated lysines (K259, K274, K281, and K311), consistent with the major influence of lysine acetylation on tau’s aggregation propensity.^58,62,64^

The C-terminal “tail” of tau (after the repeats) showed fewer modifications overall, but several are of significant interest due to their impact on tau turnover. We observed that three lysines in the tail, K369, K375, and K385, were acetylated primarily when tau homeostasis was disrupted (e.g., in kinase-treated samples or disease models). These sites were not among the earliest-reported AD acetylation marks, but they consistently appear in later-stage tauopathy samples, and their acetylation has been shown to impair chaperone-mediated tau clearance.^54^ Such modifications may interfere with the binding of tau’s C-terminus to chaperones and proteolytic machinery, thereby promoting tau accumulation under cellular stress.^65^ Beyond the three major sites, we identified several additional C-terminal PTMs. K370 was acetylated in DYRK1A- and GSK3β-treated 2N4R tau but not in untreated tau, consistent with a recently reported AD-associated site and suggesting a potentially pathology-linked modification alongside K369.^62^ K395 acetylation was detected in both control and kinase-treated samples, consistent with previous reports.^61,66^ We also observed persistent methylation of R406 in untreated and TTBK1-treated tau; to our knowledge, this is a previously unreported modification site. At the extreme C-terminus, K438 showed both acetylation and methylation in control and GSK3β-treated samples, indicating a likely constitutive and tail modification.^67^ Together, these findings expand the map of tau tail PTMs and suggest potential crosstalk between lysine acetylation and methylation in this region.^68,69^ We provide an extended analysis of lysine acetylation and arginine methylation across tau’s domains. Table S2 catalogs the novel or context-specific PTM sites identified in this study, noting the experimental conditions of their occurrence and whether they have been previously reported in human tau.

### Phosphorylation across Tau Isoforms

Mapping phosphorylation onto the 2N4R reference alignment, Figure 3A, showed that GSK3β markedly increased tau phosphorylation relative to control across all three isoforms (2N4R, 1N4R, and 0N3R), consistent with widespread hyperphosphorylation. A subset of sites remained shared between control and kinase-treated samples (blue). Most of the phosphorylation increase due to kinases was concentrated in the proline-rich region, with additional gains extending through the microtubule-binding repeats and into the C-terminal tail. As shown at the sequence level in Figure 3A, phosphorylated residues are distributed throughout the proline-rich region (residues 151–244), with substantial clustering within Ser/Thr-rich and proline-directed sequence segments. This distribution agrees with the known preference of GSK3β for Ser/Thr-rich, proline-directed sites, which are abundant in the central region of tau and overlap extensively with AD-associated phosphosites.^70^ The alignment further revealed clear isoform-specific phosphorylation patterns driven by sequence differences among tau isoforms: phosphorylation within N1 was observed only in 2N4R and 1N4R, within N2 only in 2N4R, and within R2 only in the 4R isoforms. Notably, many GSK3β-specific sites were mapped to these isoform-variable regions and to the C-terminal cluster. Together, these results show that GSK3β not only broadly hyperphosphorylates tau but also shapes distinct isoform-dependent phosphorylation patterns in regions introduced by alternative splicing.^71^ For a comprehensive review of AD-associated tau phosphorylation sites and their roles in tau aggregation, we refer readers to Supporting Information I, which compiles key phosphorylation epitopes (with sequence context and references) implicated in tau fibrillization (Table S1).

**Figure 3:**
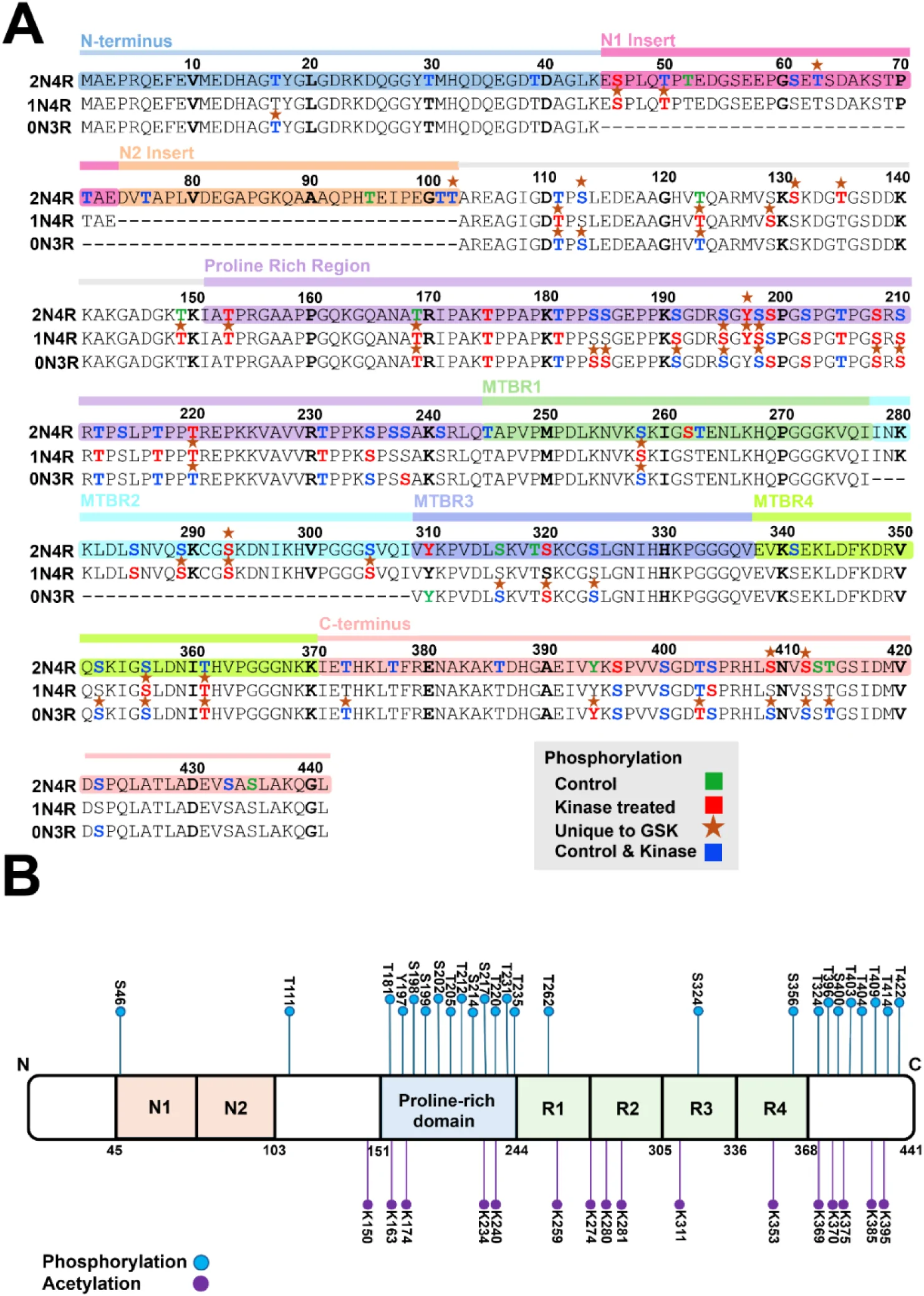
Phosphorylation and acetylation mapping across tau isoforms (2N4R, 1N4R, and 0N3R). (A) Amino-acid sequence alignment of the three tau isoforms with annotated domain boundaries. Phosphorylated residues are highlighted directly within the sequences, demonstrating their distribution and sequence context across the proline-rich region (residues 151–244 in Tau-441 numbering) and other tau domains. Green, red, and blue indicate sites detected in control samples, kinase-treated samples, and both conditions, respectively; stars indicate phosphosites detected uniquely following GSK3β treatment. (B) Domain-level map of established AD-associated phosphorylation and acetylation sites.

Figure 3B presents the 441-aa human tau 2N4R isoform as a linear domain map (N1/N2 inserts, proline-rich region, and microtubule-binding repeats R1-R4) overlaid with major pathological PTM hotspots. Blue lollipops mark phosphorylation sites concentrated in the proline-rich and C-terminal regions, including T181, S202/T205 (AT8), T231/S235, T217, and the C-terminal cluster containing S396/S404 (PHF-1) with nearby S400/S403, all widely used as markers of AD-associated tau pathology. Purple lollipops indicate acetyl-lysine sites enriched in the proline-rich and repeat domains, including K174 and K274/K280/K281, highlighting acetylation as a complementary disease-relevant PTM that can influence tau proteostasis, microtubule binding, and aggregation. Overall, the figure emphasizes the domain-specific organization of AD-relevant tau PTMs, with pathological phosphorylation enriched in the proline-rich/C-terminal regions and acetylation hotspots localized to domains involved in microtubule interaction and fibrillization. Methylation was not included in this pathology-focused panel because most methylation sites detected here lack sufficient evidence for classification as established AD-associated markers. Their inclusion in the broader PTM map (Figure 2 and Supporting Information II) is intended as a discovery resource and does not imply disease specificity.

### Improved Confidence of Phosphopeptide Identifications

The identification of phosphorylated peptides in proteomics can be tricky because the labile PTM can be lost during the MS/MS using the most widely used collision-based fragmentation methods. Additional orthogonal approaches can provide consistency support for phosphopeptide identifications. Here, we compared the predicted and experimental electrophoretic mobilities of CZE-MS/MS-detected modified peptides to assess whether their migration behavior was consistent with the assigned number of charge-altering PTMs, Figure 4. We optimized the semiempirical models reported in our previous studies^72–75^ for this specific purpose. Using the optimized semiempirical models, we observed linear correlations between the predicted and experimental mobilities of identified peptides after charge reduction, with the number of phosphorylation and acetylation PTMs as the predictor, across all CZE-MS/MS runs (R^2^ > 0.85). Figure 4 shows two examples of data from GSK3β- and TTBK1-treated 2N4R tau. Without charge correction, the predicted and experimental mobilities showed poor correlation (R^2^ < 0.65), and the phosphorylated and acetylated peptides were off the main trend. After reducing the charge by one charge unit per phosphorylation or acetylation, the linear correlation coefficients improved substantially to 0.9 or better (R^2^), and the modified peptides aligned well with the main trend. However, this mobility analysis provides peptide-level consistency support rather than independent residue-level phosphosite localization. It was not applicable to phosphopeptides detected only by nanoRPLC-MS/MS and cannot independently distinguish phosphorylation from other modifications producing a similar net charge effect. These results demonstrate the value of electrophoretic mobility analysis as an orthogonal peptide-level consistency check for CZE-MS/MS-detected modified peptides, whereas residue-level phosphosite localization remains based on the filtered HCD spectra and ptmRS probabilities.

**Figure 4:**
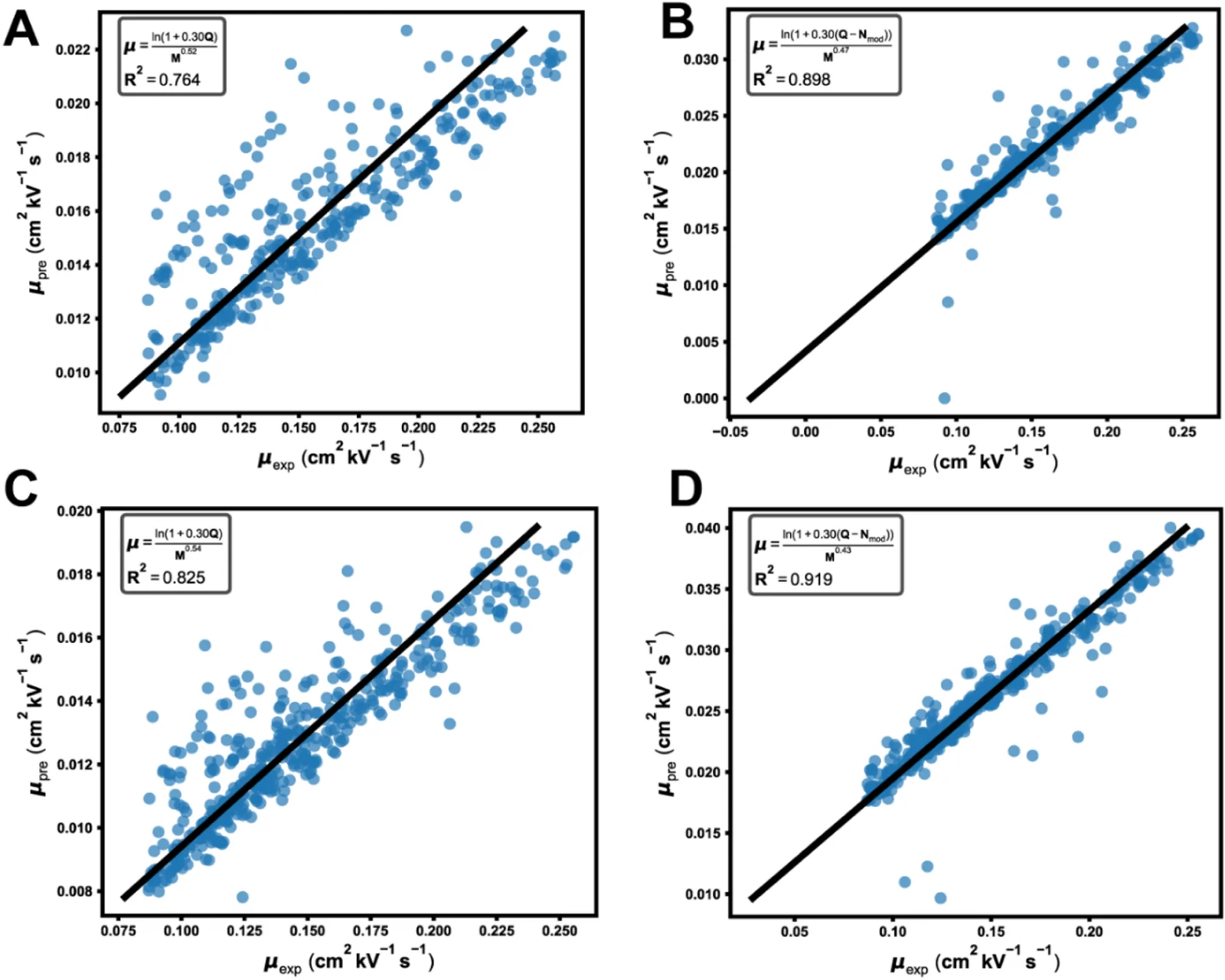
Experimental versus predicted electrophoretic mobilities of kinase-treated tau peptides before and after charge correction. (A) GSK3β-treated 2N4R tau before charge correction, (B) GSK3β-treated 2N4R tau after charge correction, (C) TTBK1-treated 2N4R tau before charge correction, and (D) TTBK1-treated 2N4R tau after charge correction. Each point corresponds to an individual tau peptide identified by CZE-MS/MS. The solid line indicates the linear fit of the data. Mobility predictions were generated using a PTM-aware model in which phosphorylation and lysine acetylation were treated as charge-reducing modifications after charge correction. The improved correlation observed after charge correction demonstrates that PTM-dependent charge adjustment enhances prediction accuracy and better captures the electrophoretic behavior of modified tau peptides.

We further provided several examples of annotated MS/MS spectra of phosphorylated peptides (Figure S1). In Figure S1A, the phosphorylated peptide IPAKTPPAPKTPPSSGEPPKSGDR (residues 171–194) was assigned as pThr181. The phosphopeptide was identified with strong search metrics (XCorr = 5.76, Percolator q = 0, PEP = 1.7 × 10^–8^, ptmRS = 100). In Figure S1B, a phosphorylated peptide, HVPGGGSVQIVYKPVDLSK (residues 299–317), was assigned as pSer305­(XCorr = 5.29, q = 0, PEP = 7.5 × 10^–7^, ptmRS = 100). The spectrum contains informative phosphate-retaining and phosphate-neutral-loss b- and y-ions, including matched y_17_ ions with and without phosphate, consistent with localization of the modification to the N-terminal portion of the peptide and supporting assignment to Ser305. For Figure S1C, the phosphorylated peptide, VQSKIGSLDNITHVPGGGNK (residues 350–369), was assigned as pSer352 (XCorr = 5.61, q = 0, PEP = 8.5 × 10^–7^, ptmRS = 100). Collectively, these spectra demonstrate that the workflow enables confident phosphosite localization across distinct tau domains, including the clinically important PRR site, Thr181, and repeat-domain sites, Ser305 and Ser352.^76^

## Conclusions

This study demonstrates that combining CZE-MS/MS with nanoRPLC-MS/MS improves residue-level characterization of tau phosphorylation and reveals kinase-specific differences not captured by a single separation method. Across the 2N4R kinase-treated data sets, we observed a shared phosphorylation core together with distinct site-selective signatures for GSK3β, TTBK1, and DYRK1A, including modification of diagnostically and mechanistically relevant residues such as Ser262 ^71^ and Ser409.^77^ These findings refine the site-resolved phosphomap of tau, highlight the analytical value of orthogonal separations for PTM discovery, and support the use of proteomics-based phosphosite patterns to distinguish convergent from kinase-specific components of tau modifications. The study also provided a useful tau PTM resource that includes various tau isoforms and kinases. We expect the tau PTM landscape revealed in this study will be helpful for further tau-focused AD studies.

## Supplementary Material





## Data Availability

The mass spectrometry proteomics data have been deposited with the ProteomeXchange Consortium via the PRIDE partner repository^78^ under the data set identifier PXD075623.

## Notes

Aggregation-associated phosphorylation sites in 2N4R tau (Supplementary Section 1); aggregation-associated phosphorylation epitopes on 2N4R Tau (Table S1); tau lysine and arginine post-translational modifications (PTMs) (Supplementary Section 2); novel or context-specific tau PTM sites identified in this study (Table S2); representative HCD CZE-MS/MS spectra of tau phosphopeptides (GSK3β-2N4R) (Figure S1); MS1-intensity comparison of representative 2N4R tau phosphopeptides (Figure S2) (PDF)
The lists of identified peptides across various conditions, protein sequence coverage summary, summary of 2N4R tau PTMs, PTM inventories across isoforms, and source data and statistical calculations for the representative phosphopeptide MS1-intensity comparison (XLSX)
