# Supplementary material for: Mapping the Landscape of Tau Post-translational Modifications and Kinase-Specific Phosphorylation Signatures by Orthogonal CZE-MS/MS and NanoRPLC-MS/MS
Source: J Proteome Res. 2026 Oct 13;25(11):6166–78. doi: 10.1021/acs.jproteome.6c00539 (PMC13750096; doi:10.1021/acs.jproteome.6c00539)
Supplement: Supplementary file 1 [file pr6c00539_si_001.pdf]

## Supporting Information I

# Mapping the Landscape of Tau Post-translational Modifications and Kinase-Specific Phosphorylation Signatures by Orthogonal CZE-MS/MS and nanoRPLC-MS/MS

Reyhane Tabatabaeian Nimavard[1], Seyed Amirhossein Sadeghi[1], Jorge A Colón Rosado[1], Yifan Yue[1], Fei Fang[1], Hsiao-Tien Chien Hagar[2], Min-Hao Kuo[2], Liangliang Sun[1*]

[1] Chemistry Dept., Michigan State University, East Lansing, Michigan, United States.

[2] Biochemistry and Molecular Biology Dept., Michigan State University, East Lansing, Michigan, United States.

* Corresponding author. Email: lsun@chemistry.msu.edu

Phone number: 517-353-0498

## Table of Content


| Topic | Page |
| --- | --- |
| Supplementary Section 1. Aggregation-Associated Phosphorylation Sites in 2N4R Tau | S3 |
| Table S1. Aggregation-Associated Phosphorylation Epitopes on 2N4R Tau | S4 |
| Supplementary Section 2. Tau Lysine and Arginine Post-Translational Modifications (PTMs) | S5-S9 |
| Table S2. Novel or Context-Specific Tau PTM Sites Identified in This Study | S10-S11 |
| Figure S1. Representative HCD CZE-MS/MS spectra of tau phosphopeptides (GSK3β-2N4R) | S12 |
| Figure S2. MS1-intensity comparison of representative 2N4R tau phosphopeptides | S13 |
| References | S14-S15 |
| The lists of identified peptides across various conditions, protein sequence coverage summary, summary of 2N4R tau PTMs, PTM inventories across isoforms, and source data and statistical calculations for the representative phosphopeptide MS1-intensity comparison. | Supporting Information II (XLSX) |

**Supplementary Section 1: Aggregation-Associated Phosphorylation Sites in 2N4R Tau.** *Detailed overview of tau phosphorylation sites linked to pathological aggregation, including a table of key phospho-epitopes (AT8, AT100, AT180, PHF-1, pS422) with supporting evidence from the literature.*

Certain phosphorylation epitopes on the longest tau isoform (2N4R tau) are strongly implicated in tau's pathological aggregation into paired helical filaments (PHFs) in Alzheimer's disease (AD). Below, we list such phosphorylation sites (or multi-site epitopes), including their tau domain, a representative peptide sequence containing the modified residue(s), and key experimental evidence linking the modification to tau aggregation. All evidence is drawn from peer-reviewed studies.

**Table S1. Aggregation-Associated Phosphorylation Epitopes on 2N4R Tau.** Each entry lists a phosphorylation site or epitope on tau, the domain of tau in which it resides, the local sequence, and a summary of evidence linking that phosphorylation to tau aggregation in AD.

| Phosphorylation Site (Epitope) | Tau Domain | Sequence Context | Evidence for Aggregation Link |
|---|---|---|---|
| **Ser202/Thr205** (AT8 epitope) | Proline-rich region | SPGSPGT | A canonical PHF-tau epitope in AD. AT8-positive tau is widely used to stage neurofibrillary pathology, and phosphorylation at S202/T205 promotes tau fibrillization, particularly when adjacent to S208. [1,2] |
| **Ser208** | Proline-rich region | TPGSRSR | Acts cooperatively with the AT8 region to enhance tau assembly. pS208 increases filament nucleation and growth and is enriched in mature tangles. [1,3] |
| **Thr212/Ser214** (AT100 epitope; often including Thr217) | Proline-rich region | SRTPSLPTP | A PHF-associated epitope linked to advanced pathological tau. Phosphorylation at T212/S214 is associated with tau filament formation and aggregation-prone tau states.[3,4] |
| **Thr231/Ser235** (AT180 epitope) | Proline-rich region | VRTPPKSP | An early pathological phospho-epitope that weakens tau–microtubule interactions and favors misfolding and aggregation. pT231 is strongly associated with PHF-tau.[1,4] |
| **Ser396/Ser404** (PHF-1 epitope) | C-terminal region | KSPVVSGDTS | A late-stage C-terminal phospho-epitope highly enriched in AD filaments. Phosphorylation at S396/S404 strongly enhances tau fibrillization and is a classic marker of aggregated tau.[5] |
| **Ser422** | C-terminal region | VDSPQLA | A late-stage aggregation-associated site linked to tau oligomerization, neurofibrillary tangles, and cognitive decline. pS422 is associated particularly with stabilized oligomeric/aggregated tau species.[6] |

**Note:** All sites listed above are on human 2N4R tau (441 amino acids) and have been implicated by experimental evidence as promoters of tau assembly in the context of AD. Notably, some other tau

phospho-sites (e.g., Ser262 in the repeat domain) do not promote aggregation; in fact, Ser262 phosphorylation can inhibit tau filament formation, and thus are not included here. The phosphorylation epitopes highlighted (AT8, Ser208, AT100, AT180, PHF-1, pS422) collectively contribute to the aberrant “hyperphosphorylated” state of tau that drives its self-assembly into the insoluble neurofibrillary tangles characteristic of Alzheimer’s disease.

**Supplementary Section 2: Tau Lysine and Arginine Post-Translational Modifications (PTMs).** *Comprehensive analysis of tau lysine acetylation and arginine methylation sites across tau domains (N-terminal, proline-rich, repeat, and C-terminal regions), highlighting newly identified or context-specific modifications. A summary table of novel PTM sites observed under specific conditions is included.*

In this section, we provide a detailed survey of tau lysine and arginine PTMs identified in our study, organized by tau’s domain structure. We describe acetylation (on Lys residues) and methylation (on Lys or Arg residues) detected in each major domain of tau the N-terminal domain, the proline-rich region, the microtubule-binding repeat domain, and the C-terminal tail noting which sites appear to be constitutive, which are induced only under specific conditions (such as *in vitro* kinase treatments or in certain isoforms), and which correspond to known pathological modifications. A summary table of novel or context-specific PTM sites is provided at the end of this section.

The N-terminal “projection” domain of tau harbored numerous modifications, predominantly on lysines within or adjacent to the two N-terminal inserts. Arg23 and Lys24, located in the extreme N-terminus (encoded by exon 1), were consistently methylated in our data, and Lys24 also underwent acetylation in both control human brain tau and GSK3β-treated tau. These methylation marks were observed across all isoforms (0N, 1N, and 2N), indicating that they are constitutive rather than disease-specific modifications. No prior literature has implicated R23 or K24 in tauopathies (K24 modifications have been reported only very rarely). Lys44, another exon 1 residue present in all isoforms, likewise showed both acetylation and methylation in control and kinase-treated samples. This site has been observed sporadically in previous studies (for example, methylated K44 in CBD tau fibrils and occasional acetyl-K44 have been reported[7]), but it has not been documented to play a role in tau pathology.

In contrast, modifications on residues within the N-terminal inserts were highly isoform-dependent. For example, Lys67 (in the first N-terminal insert, present only in the 1N and 2N isoforms) was acetylated exclusively after GSK3β treatment of 2N4R tau and was methylated in CDK-treated 1N4R tau, but it was completely absent in 0N tau (which lacks that insert). Similarly, Lys87 (within the second insert, unique to 2N4R) became acetylated only under GSK3β treatment. These insert-region marks were not detected in control tau and have no precedent in AD studies. To our knowledge, neither K67 nor K87 has been reported in the literature, suggesting that such insert-specific modifications arise from experimental manipulations rather than from physiological or pathological processes (see **Table S2** for details on these sites).

We also observed several clustered lysine modifications in the distal N-terminus (after the insert regions). Lys130, Lys132, Lys140, Lys141, and Lys143 were each acetylated in this mid-region (often accompanied by parallel methylation). For instance, K132 was acetylated even in untreated 2N4R tau and persisted (with occasional methylation) across all kinase conditions and isoforms, indicating that it is consistently modified at baseline. Aside from K132 (which had previously been identified as a methylation site in normal human brain tau)[8]. None of these mid-N-terminal sites has been reported in prior studies. We therefore consider K130, K140, K141, and K143 to be novel N-terminal PTMs. (Lys150, a known pathological acetylation site at the border of this domain, is described in the main Discussion of the manuscript.)

Within the proline-rich region, we identified both novel modifications and modifications that overlap with known disease-associated sites. Lys164, which neighbors the well-known site K163, was acetylated only in 1N4R tau under CDK treatment. This K164 modification is novel, occurring in an isoform-specific context and not previously reported in the literature.

Multiple arginine methylation events were mapped in the proline-rich region. Arg155 was found to be methylated in GSK3β-treated 2N4R tau. This PTM is noteworthy because R155 methylation has been observed in both normal and AD tau[8,9], indicating that it is a constitutive rather than disease-specific modification. Similarly, Arg170 and Arg194 were found to be methylated (in both untreated and kinase-treated tau). To our

knowledge, methylation at R170 and R194 has not been reported previously. Their presence across both control and kinase conditions suggests that they are not driven by tau hyperphosphorylation or aggregation, but instead are part of tau's normal PTM landscape. Two additional arginine residues, R209 and R211, were identified as methylated exclusively under kinase-treated conditions and were not detected in control tau. R209 methylation was observed only with TTBK1, whereas R211 methylation was detected with both TTBK1 and GSK3β in 2N4R and 1N4R tau, consistent with the idea that kinase-dependent phosphorylation states may facilitate arginine methylation at these positions. To our knowledge, neither site has been previously reported to be methylated in tau, indicating that R209 and R211 are candidate novel arginine PTMs. The proline-rich domain also contained numerous lysine modifications, including several that overlap with previously reported AD-related sites. Lys224 and Lys225, positioned immediately C-terminal to the polyproline motifs, were detected as acetylated in a condition- and isoform-dependent manner. K224 acetylation was observed in untreated 0N3R tau, whereas K225 acetylation was identified in control, GSK3β-treated, and TTBK1-treated 2N4R tau and in GSK3β-treated 0N3R tau. To our knowledge, neither residue has been previously reported as an acetylation site in tau, indicating that K224 and K225 represent candidate novel tau acetylation sites. In contrast, Lys234 and Lys240 were acetylated in nearly all samples; those residues are established AD-associated acetylation sites,[10] confirming that while they can be modified under normal conditions, their pronounced acetylation occurs chiefly in disease.[10,11] The prominent acetylation site Lys274, located at the junction with the repeat domain, was not detected in normal tau and appeared only minimally in kinase-treated tau, underscoring its disease specificity.[12]

The repeat domain (comprising the four microtubule-binding repeats, R1-R4, in 2N4R tau) exhibited a wide range of PTMs. Our data suggests that acetylation of Lys281 can occur in normal tau, even though K281 acetylation is far more pronounced in tauopathies. By contrast, Lys274 and Lys280 (under CDK treatment in 0N3R) remained unmodified in our control samples, reinforcing that their acetylation is restricted to disease contexts.[10,12,13]

Several modifications in the later repeats (the R3-R4 region) were also observed. Lys321 (at the junction between R3 and R4) was acetylated in both control and GSK3β-treated 2N4R tau. This site has been reported to be acetylated under physiological conditions (regulated by HDAC6[14]), although it has not been highlighted in disease-centric studies. Lys331 (mid-R4) was acetylated in control tau and was additionally acetylated (and even methylated) in kinase-treated 0N3R and 2N4R tau. This site has not been reported in prior literature, making it a newly identified acetylation site. Two lysines just after the repeat region, Lys347 and Lys353, also showed evidence of modification. We detected acetylation at these sites only in limited instances (for example, K347 acetylation in one GSK3β-treated 2N4R sample, and K353 acetylation in all control isoforms as well as in GSK3β-treated 0N3R and 2N4R tau). Acetylation at K347 was recently discovered *in vivo*[10], and K353 is a well-established site (present on tau in healthy human brains and observed in acetylated form in AD tau[10]). Even a slight modification at K347 or K353 could plausibly weaken tau's affinity for microtubules, though direct evidence for this effect is lacking.

*Major pathological acetylation sites in the repeat domain (including K259, K274, K281, K311) are described in the main Discussion of the manuscript.*

The C-terminal "tail" of tau (residues 369-441, following the repeat region) contained fewer PTMs overall, but several of these are of great interest given emerging evidence that C-terminal acetylation can influence tau clearance.[15,16] Three lysines in the C-terminal tail, K369, K375, and K385, were observed to be acetylated across both control and kinase-perturbed conditions, supporting the idea that they are important sites within the tau PTM landscape.[10,15] Beyond these sites, the C-terminus yielded a few additional PTMs of note. Lys370 (immediately adjacent to K369) was acetylated in DYRK1A-treated and GSK3β-treated 2N4R tau. We did not observe K370 in untreated 2N4R tau, and this site had not been highlighted in prior work. However, K370 has been detected as an acetylated residue in AD brain tissue in a recent proteomic.[10] Thus, K370 appears to be a newly identified tau acetylation site that may be modified preferentially under pathological conditions, alongside the nearby K369.

We also observed persistent methylation of Arg406 in control tau (untreated 0N3R and 2N4R) and in TTBK1-treated 2N4R tau. (Notably, the aggregation-prone hexapeptide motif **VEVKSE**, also known as PHF6, spanning residues 337-342 in the fourth repeat, is a driver of tau fibril formation; R406 lies just downstream of this motif, in the C-terminal tail region.) To our knowledge, arginine methylation at R406 has not been reported previously on tau; we report it here as a novel modification, though its functional implications remain uncertain. Finally, Lys438 at the extreme C-terminus was observed in both methylated and acetylated states. Methylation at K438 was detected in control 2N4R tau, control 0N3R tau, and GSK3β-treated 0N3R tau, whereas acetylation at this residue was identified in control 2N4R tau. To our knowledge, K438 has not been previously described as a modified tau lysine, supporting its assignment as a novel C-terminal PTM site. The presence of K438 modification in control samples, together with its detection across more than one condition, suggests that this residue may represent a basally modified or constitutively accessible PTM site, rather than a strictly kinase-induced event.*(The acetylation of K369, K375, and K385 and potential crosstalk between acetylation and methylation at these sites are discussed in the main text of the manuscript.)*

**Table S2. Novel or context-specific tau PTM sites were identified in this study.** This table lists tau post-translational modification sites that were not previously reported in the literature or that were observed only under specific conditions (e.g., only in *in vitro* kinase treatments or in particular tau isoforms). For each site, the residue and modification type(s) are given, along with the sample/condition in which the site was detected in our experiments and its status in the literature.

| **Residue** | **Modification Type(s)** | **Literature Status** |
|---|---|---|
| R23 | Methylation | Novel. To our knowledge, this site has not been reported in the literature previously (for either human brain or *E. coli* tau). |
| K67 | Acetylation; Methylation | Novel. To our knowledge, this site has not been reported in the literature previously (for either human brain or *E. coli* tau). |
| K87 | Acetylation | Novel. To our knowledge, this site has not been reported in the literature previously (for either human brain or *E. coli* tau). |
| K130 | Acetylation; Methylation | Novel. Not reported in prior tau studies. |
| K140 | Acetylation; Methylation | Novel. Not reported in prior tau studies. |
| K141 | Acetylation | Novel. Not reported in prior tau studies. |
| K143 | Acetylation; Methylation | Novel. Not reported in prior tau studies. |
| K148 | Acetylation | Novel. To our knowledge, this site has not been reported previously in the literature. |
| K164 | Acetylation | Novel. Not reported in prior literature. |
| R170 | Methylation | Novel. Not reported in prior literature. |

| Residue | Modification Type(s) | Literature Status |
|---|---|---|
| R194 | Methylation | Novel. Not reported in prior literature. |
| R209 | Acetylation; Methylation | Novel. Not reported in prior literature. |
| R211 | Methylation | Novel. Not reported in prior literature. |
| K224 | Acetylation | Novel. Not reported in prior literature. |
| K225 | Acetylation | Novel. Not reported in prior literature. |
| K268 | Methylation (putative) | Novel. To our knowledge, this site has not been reported previously. |
| K298 | Acetylation (putative) | Novel. To our knowledge, this site has not been reported previously. |
| K331 | Acetylation; Methylation | Novel. Not reported in prior literature. |
| K383 | Acetylation | Novel. Not reported in prior literature. |
| R406 | Methylation | Novel. To our knowledge, this site has not been reported previously in the literature. |
| K438 | Acetylation; Methylation | Novel. To our knowledge, this site has not been reported in the literature previously (for either human brain or *E. coli* tau). |

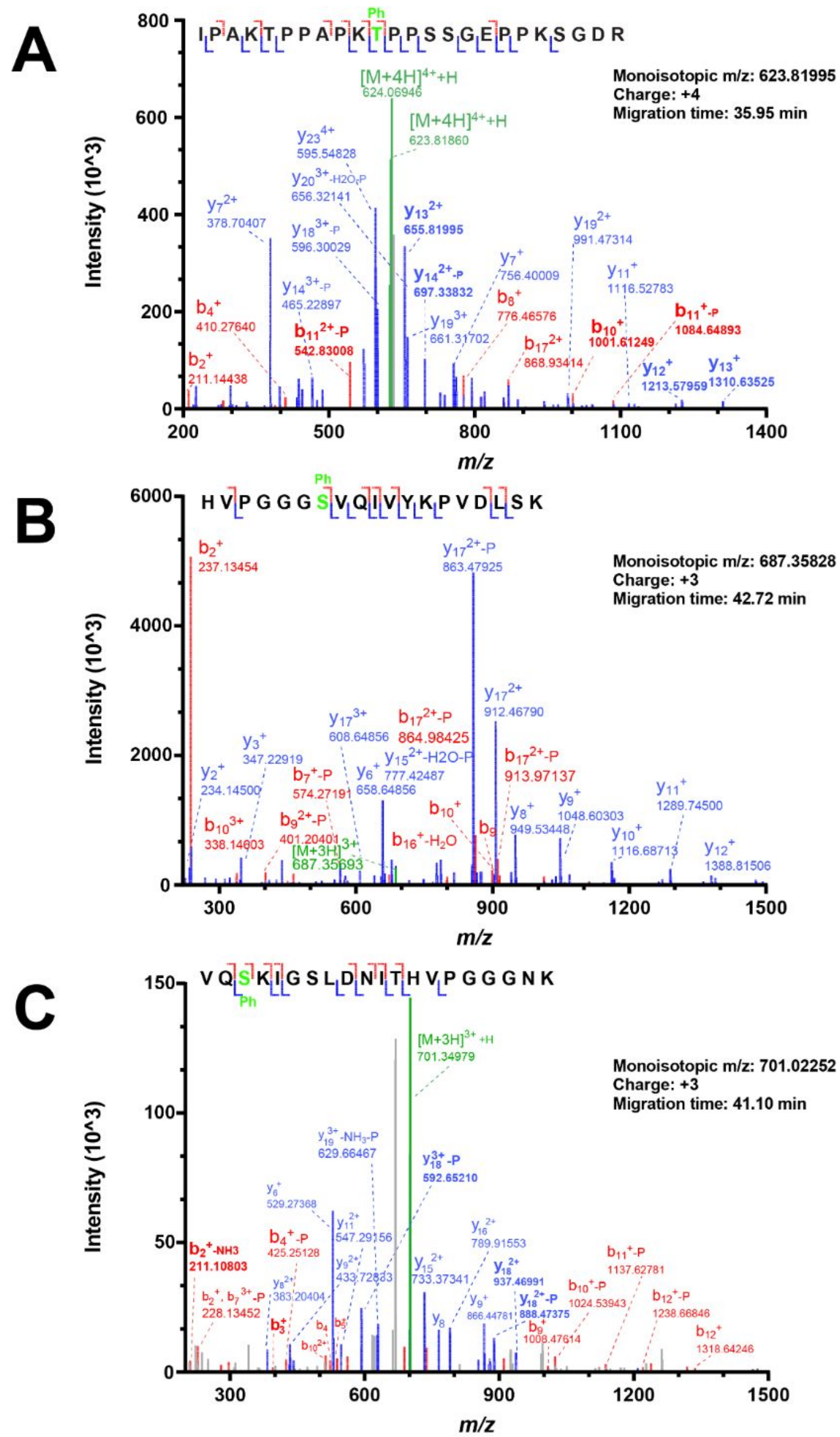


**Figure S1.** Representative HCD CZE-MS/MS spectra of tau phosphopeptides (GSK3β-2N4R) showing residue-level phosphorylation site localization. Annotated b- and y-ion series support localization of phosphorylation in peptides from the proline-rich region (A) and microtubule-binding repeat domain (B, C), identifying pThr181, pSer305, and pSer352, respectively.

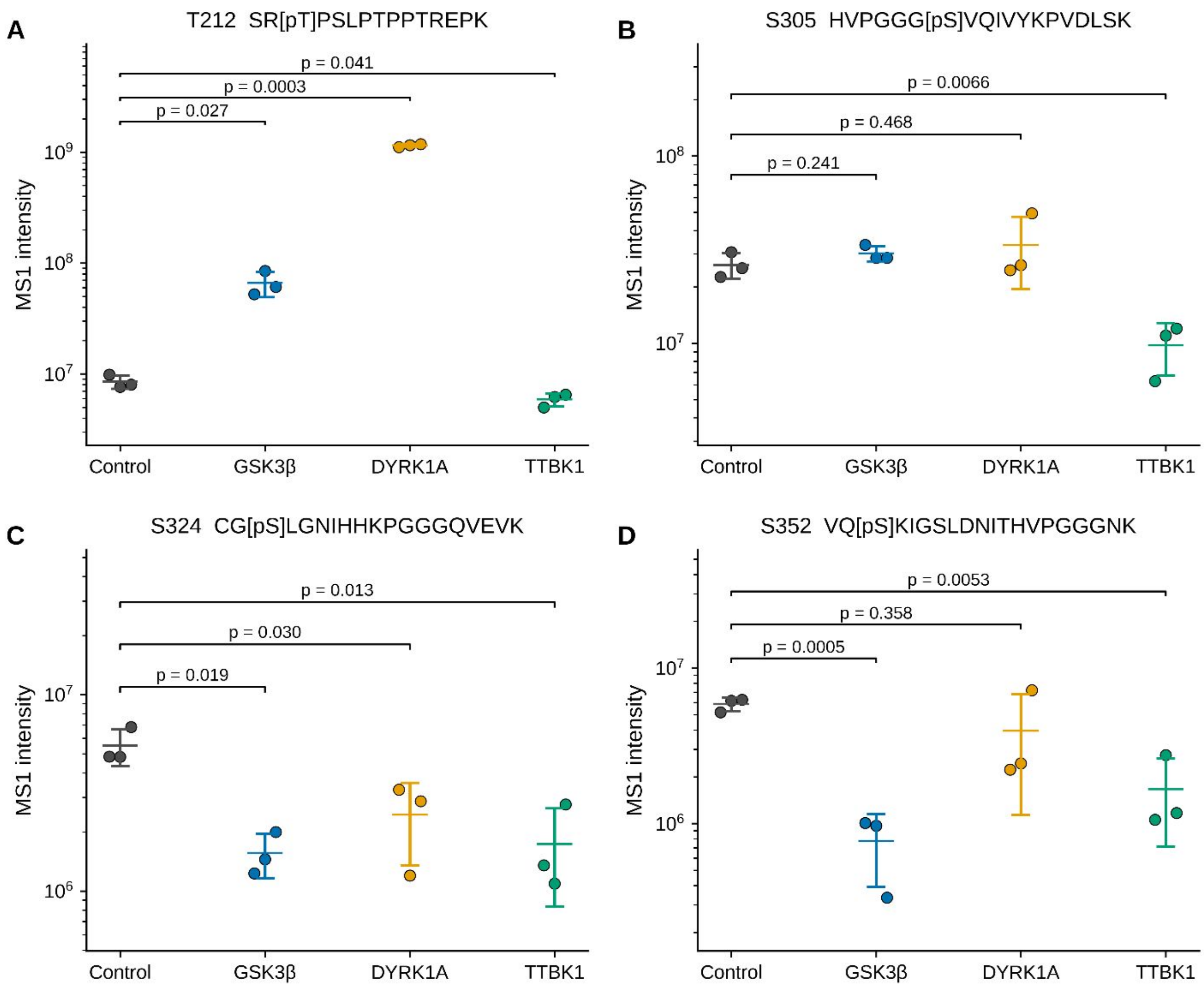


**Figure S2.** MS1 intensities of representative 2N4R tau phosphopeptides across untreated and kinase-treated conditions. Raw MS1 intensities were manually extracted from the corresponding raw CZE-MS/MS data. Each point represents one technical replicate (n = 3 per condition), and error bars represent mean ± SD. The y-axis is displayed on a logarithmic scale for visualization. Each kinase-treated condition was compared with the untreated control using a two-tailed Welch's unpaired t-test performed on the untransformed raw MS1 intensities; unadjusted p-values are shown.

## References

(1) Despres, C.; Byrne, C.; Qi, H.; Cantrelle, F. X.; Huvent, I.; Chambraud, B.; Baulieu, E. E.; Jacquot, Y.; Landrieu, I.; Lippens, G.; Smet-Nocca, C. Identification of the Tau Phosphorylation Pattern That Drives Its Aggregation. *Proceedings of the National Academy of Sciences of the United States of America*. National Academy of Sciences August 22, **2017**, pp 9080–9085. https://doi.org/10.1073/pnas.1708448114.

(2) Braak, H.; Braak, E.; Braak, E. *Staging of Alzheimer's Disease-Related Neurofibrillary Changes*; **1995**; Vol. 16.

(3) Xia, Y.; Xia, Y.; Prokop, S.; Prokop, S.; Prokop, S.; Prokop, S.; Gorion, K. M. M.; Gorion, K. M. M.; Kim, J. D.; Kim, J. D.; Sorrentino, Z. A.; Sorrentino, Z. A.; Bell, B. M.; Bell, B. M.; Manaois, A. N.; Manaois, A. N.; Chakrabarty, P.; Chakrabarty, P.; Chakrabarty, P.; Davies, P.; Giasson, B. I.; Giasson, B. I.; Giasson, B. I. Tau Ser208 Phosphorylation Promotes Aggregation and Reveals Neuropathologic Diversity in Alzheimer's Disease and Other Tauopathies. *Acta Neuropathol. Commun.* **2020**, *8* (1). https://doi.org/10.1186/s40478-020-00967-w.

(4) Alonso, A. D.; Di Clerico, J.; Li, B.; Corbo, C. P.; Alaniz, M. E.; Grundke-Iqbal, I.; Iqbal, K. Phosphorylation of Tau at Thr212, Thr231, and Ser262 Combined Causes Neurodegeneration. *Journal of Biological Chemistry* **2010**, *285* (40), 30851–30860. https://doi.org/10.1074/jbc.M110.110957.

(5) Liu, M.; Sui, D.; Dexheimer, T.; Hovde, S.; Deng, X.; Wang, K. W.; Lin, H. L.; Chien, H. T.; Kweon, H. K.; Kuo, N. S.; Ayoub, C. A.; Jimenez-Harrison, D.; Andrews, P. C.; Kwok, R.; Bochar, D. A.; Kuret, J.; Fortin, J.; Tsay, Y. G.; Kuo, M. H. Hyperphosphorylation Renders Tau Prone to Aggregate and to Cause Cell Death. *Mol. Neurobiol.* **2020**, *57* (11), 4704–4719. https://doi.org/10.1007/s12035-020-02034-w.

(6) Tiernan, C. T.; Combs, B.; Cox, K.; Morfini, G.; Brady, S. T.; Counts, S. E.; Kanaan, N. M. Pseudophosphorylation of Tau at S422 Enhances SDS-Stable Dimer Formation and Impairs Both Anterograde and Retrograde Fast Axonal Transport. *Exp. Neurol.* **2016**, *283*, 318–329. https://doi.org/10.1016/j.expneurol.2016.06.030.

(7) Tarutani, A.; Kametani, F.; Tahira, M.; Saito, Y.; Yoshida, M.; Robinson, A. C.; Mann, D. M. A.; Murayama, S.; Tomita, T.; Hasegawa, M. Distinct Tau Folds Initiate Templated Seeding and Alter the Post-Translational Modification Profile. *Brain* **2023**, *146* (12), 4988–4999. https://doi.org/10.1093/brain/awad272.

(8) Huseby, C. J.; Hoffman, C. N.; Cooper, G. L.; Cocuron, J. C.; Alonso, A. P.; Thomas, S. N.; Yang, A. J.; Kuret, J. Quantification of Tau Protein Lysine Methylation in Aging and Alzheimer's Disease. *Journal of Alzheimer's Disease* **2019**, *71* (3), 979–991. https://doi.org/10.3233/JAD-190604.

(9) Bichmann, M.; Prat Oriol, N.; Ercan-Herbst, E.; Schöndorf, D. C.; Gomez Ramos, B.; Schwärzler, V.; Neu, M.; Schlüter, A.; Wang, X.; Jin, L.; Hu, C.; Tian, Y.; Ried, J. S.; Haberkant, P.; Gasparini, L.; Ehrnhoefer, D. E. SETD7-Mediated Monomethylation Is Enriched on Soluble Tau in Alzheimer's Disease. *Mol. Neurodegener.* **2021**, *16* (1). https://doi.org/10.1186/s13024-021-00468-x.

(10) Wesseling, H.; Mair, W.; Kumar, M.; Schlaffner, C. N.; Tang, S.; Beerepoot, P.; Fatou, B.; Guise, A. J.; Cheng, L.; Takeda, S.; Muntel, J.; Rotunno, M. S.; Dujardin, S.; Davies, P.; Kosik, K. S.; Miller, B. L.; Berretta, S.; Hedreen, J. C.; Grinberg, L. T.; Seeley, W. W.; Hyman, B. T.; Steen, H.; Steen, J. A. Tau PTM Profiles Identify Patient Heterogeneity and Stages of Alzheimer's Disease. *Cell* **2020**, *183* (6), 1699-1713.e13. https://doi.org/10.1016/j.cell.2020.10.029.

(11) Chakraborty, P.; Rivière, G.; Hebestreit, A.; de Opakua, A. I.; Vorberg, I. M.; Andreas, L. B.; Zweckstetter, M. Acetylation Discriminates Disease-Specific Tau Deposition. *Nat. Commun.* **2023**, *14* (1). https://doi.org/10.1038/s41467-023-41672-1.

(12) Tracy, T. E.; Sohn, P. D.; Minami, S. S.; Wang, C.; Min, S. W.; Li, Y.; Zhou, Y.; Le, D.; Lo, I.; Ponnusamy, R.; Cong, X.; Schilling, B.; Ellerby, L. M.; Huganir, R. L.; Gan, L. Acetylated Tau Obstructs KIBRA-Mediated Signaling in Synaptic Plasticity and Promotes Tauopathy-Related Memory Loss. *Neuron* **2016**, *90* (2), 245–260. https://doi.org/10.1016/j.neuron.2016.03.005.

(13) Irwin, D. J.; Cohen, T. J.; Grossman, M.; Arnold, S. E.; McCarty-Wood, E.; Van Deerlin, V. M.; Lee, V. M. Y.; Trojanowski, J. Q. Acetylated Tau Neuropathology in Sporadic and Hereditary Tauopathies. *American Journal of Pathology* **2013**, *183* (2), 344–351. https://doi.org/10.1016/j.ajpath.2013.04.025.

(14) Trzeciakiewicz, H.; Ajit, D.; Tseng, J. H.; Chen, Y.; Ajit, A.; Tabassum, Z.; Lobrovich, R.; Peterson, C.; Riddick, N. V.; Itano, M. S.; Tripathy, A.; Moy, S. S.; Lee, V. M. Y.; Trojanowski, J. Q.; Irwin, D. J.; Cohen, T. J. An HDAC6-Dependent Surveillance Mechanism Suppresses Tau-Mediated Neurodegeneration and Cognitive Decline. *Nat. Commun.* **2020**, *11* (1). https://doi.org/10.1038/s41467-020-19317-4.

(15) Alquezar, C.; Schoch, K. M.; Geier, E. G.; Ramos, E. M.; Scrivo, A.; Li, K. H.; Argouarch, A. R.; Mlynarski, E. E.; Dombroski, B.; Deture, M.; Dickson, D. W.; Yokoyama, J. S.; Cuervo, A. M.; Burlingame, A. L.; Schellenberg, G. D.; Miller, T. M.; Miller, B. L.; Kao, A. W. *TSC1 Loss Increases Risk for Tauopathy by Inducing Tau Acetylation and Preventing Tau Clearance via Chaperone-Mediated Autophagy*; **2021**; Vol. 7.

(16) Holthoff, V. A.; Ferris, S.; Ihl, R.; Robert, P.; Winblad, B.; Gauthier, S.; Sternberg, K.; Tennigkeit, F. Validation of the Relevant Outcome Scale for Alzheimer's Disease: A Novel Multidomain Assessment for Daily Medical Practice. *Alzheimer's Res. Ther.* **2011**, *3* (5). https://doi.org/10.1186/alzrt89.
